# Efficacy comparison of toripalimab versus pembrolizumab in neoadjuvant treatment of breast cancer: impact of chemotherapy regimen and sequence on pathologic complete response

**DOI:** 10.3389/fonc.2025.1699963

**Published:** 2025-12-19

**Authors:** Yunjiao Zhang, Bo Zhang, Xi Chen, Xue Zhang, Chutuo Liu, Zhe Wang, Rui Ling

**Affiliations:** 1 Department of Thyroid, Breast and Vascular Surgery, Xijing Hospital, Fourth Military Medical University, Xi’an, Shaanxi, China; 2 Department of General Surgery, The First Affiliated Hospital of Soochow University, Suzhou, Jiangsu, China

**Keywords:** breast cancer, neoadjuvant therapy, immune checkpoint inhibitors, toripalimab, pembrolizumab, pathologic complete response, real-world study

## Abstract

**Objective:**

This real-world study aimed to compare the efficacy and safety of the domestically developed PD-1 inhibitor toripalimab with pembrolizumab in the neoadjuvant treatment of breast cancer, and to further investigate the impact of treatment cycles, platinum-based chemotherapy, and chemotherapy sequencing on pathologic complete response (pCR).

**Methods:**

This retrospective study included 114 breast cancer patients who received neoadjuvant therapy with either toripalimab or pembrolizumab at the First Affiliated Hospital of Air Force Medical University between January 2021 and January 2025. Participants were stratified into groups based on: (1) immunotherapy cycles (completed 8 cycles vs. incomplete); (2) chemotherapy regimen (platinum vs. non-platinum); (3) chemotherapy sequence (EC-T, EC-TCb, T-EC, TCb-EC, or other); and (4) PD-1 inhibitor type (toripalimab vs. pembrolizumab). The primary endpoint was total pCR (tpCR; ypT0/is ypN0). Secondary endpoints included breast pCR (bpCR) and residual cancer burden (RCB). Statistical analyses incorporated Chi-square tests and Fisher’s exact tests for group comparisons. Baseline characteristics were compared across groups to assess the potential for confounding.

**Results:**

No significant differences were observed in tpCR (56.7% vs. 46.8%; P=0.297) or RCB-0 rates (56.7% vs. 44.7%; P=0.524) between patients completing versus not completing 8 immunotherapy cycles. The tpCR rate did not differ significantly between platinum-based and non-platinum-based regimens (53.6% vs. 51.7%, P=0.843). Chemotherapy sequence significantly affected efficacy: the TCb-EC group achieved the highest tpCR rate (79.2%), significantly outperforming EC-T (56.4%) and other regimens (27.3%). Toripalimab and pembrolizumab showed no significant difference in tpCR rate (56.8% vs. 50.0%, P=0.777) or RCB-0 rate (56.8% vs. 48.6%, P=0.903).

**Conclusion:**

Toripalimab demonstrated non-inferior efficacy compared to pembrolizumab in the neoadjuvant treatment of breast cancer. Chemotherapy sequence—particularly the TCb-EC regimen—significantly predicted pCR, while immunotherapy cycle number and platinum use did not influence pathologic outcomes. These real-world findings support toripalimab’s clinical use and underscore the importance of optimizing chemoimmunotherapy sequencing.

## 1 Introduction

Breast cancer remains the most commonly diagnosed malignancy among women worldwide (1). Its high heterogeneity necessitates precision treatment strategies based on molecular subtypes, such as hormone receptor (HR) status and human epidermal growth factor receptor 2 (HER2) status (2). Neoadjuvant chemotherapy (NACT) has become a standard of care for locally advanced or high-risk early-stage breast cancer (3, 4). The objectives of NACT encompass tumor downstaging, enhancing the feasibility of breast-conserving surgery, and enabling an *in vivo* assessment of treatment sensitivity through pathologic complete response (pCR), which serves as a robust surrogate endpoint for long-term survival outcomes such as event-free survival (EFS) (5).

Recent progress in cancer immunotherapy, particularly the emergence of inhibitors targeting the programmed death-1 (PD-1) receptor and its ligand (PD-L1), has revolutionized the systemic treatment landscape for breast cancer (6). Initial breakthroughs were achieved in triple-negative breast cancer (TNBC), where landmark Phase III trials such as KEYNOTE-522 demonstrated that the addition of pembrolizumab to chemotherapy significantly improved pathologic complete response (pCR) rates and extended event-free survival (EFS) in the neoadjuvant and adjuvant treatment of early, high-risk TNBC, thereby establishing immune-chemocombination as a standard therapeutic approach in this population (7, 8). Subsequent research has expanded to other subtypes. For instance, the KEYNOTE-756 trail revealed that pembrolizumab combined with chemotherapy also significantly increased pCR rates in patients with high-risk early-stage hormone receptor-positive (HR+)/HER2-negative breast cancer (9). Furthermore, based on the KEYNOTE-355 results, pembrolizumab also plays a key role in the first-line treatment of advanced PD-L1-positive TNBC (10). These advances underscore the expanding applicability of immunotherapy across diverse breast cancer subtypes.

Despite the notable efficacy and widespread clinical adoption of pembrolizumab, its substantial cost and challenges in accessibility remain significant barriers. This is particularly true in settings with healthcare resource disparities, where these factors often preclude timely treatment for many patients (11). Toripalimab, a PD-1 inhibitor independently developed in China, has demonstrated compelling antitumor activity and a manageable safety profile across multiple solid tumors, leading to its approval for several indications (12). In breast cancer, emerging evidence, such as that from the NeoTENNIS study, supports the promising efficacy and safety of toripalimab in the neoadjuvant management of TNBC (13). Nevertheless, despite its increasing integration into clinical practice, direct comparative evidence between toripalimab and pembrolizumab remains limited across different molecular subtypes of breast cancer. In the absence of head-to-head randomized controlled trials (RCTs), well-conducted retrospective real-world studies are essential to inform clinical decision-making and guide resource-efficient therapeutic strategies.

Therefore, this study utilized real-world data from patients with multiple molecular subtypes of breast cancer who received neoadjuvant therapy incorporating either toripalimab or pembrolizumab. Group comparisons and univariate statistical tests were applied to assess the association between key variables and treatment outcomes. We specifically examined the potential influence of several clinically relevant variables, including completion of treatment cycles, administration of platinum-based chemotherapy, and sequencing of chemotherapeutic agents. The findings offer critical evidence to guide clinicians in selecting PD-1 inhibitors and support the rational use of effective, more accessible domestically developed innovative drugs across all breast cancer subtypes.

## 2 Materials and methods

### 2.1 Study design and patient cohort

This single-center, retrospective, observational study was conducted among breast cancer patients who received anti-PD1 neoadjuvant therapy at the Department of Thyroid, Breast, and Vascular Surgery, First Affiliated Hospital of Air Force Medical University, from January 2021 and January 2025. Eligible patients were required to meet the following inclusion criteria: (1) pathologically confirmed invasive breast cancer; (2) treatment with a neoadjuvant combination regimen containing either pembrolizumab or toripalimab; (3) completion of all planned neoadjuvant therapy followed by radical surgery; and (4) availability of complete neoadjuvant treatment documentation and postoperative pathological assessment. Exclusion criteria comprised: (1) a history of other active malignancies; (2) alteration of the initially planned immunotherapy or chemotherapy regimen during neoadjuvant treatment for any reason; and (3) incomplete clinical or pathological data. A total of 114 patients were included in the final analysis. All data were retrospectively collected from the institutional electronic medical record (EMR) system.

### 2.2 Treatment regimens and grouping

Treatment plans for all patients were formulated by a multidisciplinary team (MDT) according to domestic and international guidelines and clinical practice. Based on differences in treatment regimens, patients were grouped for comparison along the following four dimensions:

1. Anti-PD-1 treatment cycles: Divided into completed 8 cycles group (Full 8-cycle group) and did not complete 8 cycles group (<8 cycles group).

2. Chemotherapy regimen: Primarily compared platinum-based regimens vs. non-platinum-based regimens.

3. Chemotherapy sequence: Based on clinically adopted schemes, patients were divided into five groups:

EC-T group: Received epirubicin + cyclophosphamide (EC) for 4 cycles, followed by paclitaxel (T) for 4 cycles.
EC-TCb group: Received EC for 4 cycles, followed by paclitaxel + carboplatin (TCb) for 4 cycles.
T-EC group: Received paclitaxel (T) for 4 cycles, followed by EC for 4 cycles.
TCb-EC group: Received paclitaxel + carboplatin (TCb) for 4 cycles, followed by EC for 4 cycles.
Other regimens group: Received other chemotherapy schemes outside the four standard sequential patterns mentioned above (e.g., single-agent taxane, platinum-containing but with different cycle numbers).

4. Type of anti-PD-1 inhibitor: Divided into toripalimab group (240 mg, every 3 weeks) and pembrolizumab group (200 mg, every 3 weeks).

### 2.3 Efficacy evaluation

All patients underwent radical surgery approximately 4–6 weeks after completing neoadjuvant therapy. Postoperative surgical specimens were independently evaluated by two senior pathologists blinded to the clinical treatment groups.

Primary efficacy endpoint: Total pathologic complete response (tpCR): Absence of invasive cancer in both the breast primary and axillary lymph nodes (allowance for residual ductal carcinoma *in situ*, DCIS), i.e., ypT0/is and ypN0.

Secondary efficacy endpoints:

Breast pathologic complete response (bpCR): Absence of invasive cancer in the breast primary only (allowance for DCIS), regardless of axillary lymph node status, i.e., ypT0/is (any ypN status).

Residual Cancer Burden (RCB): Calculated according to the standard formula from MD Anderson Cancer Center, comprehensively evaluating the area of the primary cancer bed, the proportion of cancer cells, the number and diameter of lymph node metastases, etc. Patients were classified as RCB-0 (pCR), RCB-I (minimal residual), RCB-II (moderate residual), or RCB-III (extensive residual).

### 2.4 Statistical analysis

Data were analyzed using IBM SPSS Statistics 26.0 or R software (version 4.2.1). Categorical variables were expressed as frequency (n) and percentage (%). Intergroup comparisons were performed using the Chi-square test or Fisher’s exact test (if theoretical frequency <5). A two-sided P-value <0.05 was considered statistically significant.

## 3 Results

### 3.1 Impact of immunotherapy cycle completion on pathologic response rates

This study first assessed the impact of completing the planned immunotherapy cycles on efficacy. Patients were divided into those who completed 8 cycles (N=67) and those who did not (<8 cycles group, N=47). A systematic comparison of clinicopathological characteristics and treatment responses between the two groups was conducted (**Table 1**). The results showed that the two groups were well-balanced in baseline clinicopathological characteristics. The choice of anti-PD-1 drug (P=0.737) and the use of platinum-based regimens (P=0.427) were not significantly different between the groups. Baseline characteristics such as age distribution (P=0.677), menopausal status (P=0.186), tumor T stage (P=0.117), and nodal status (P=0.595) also showed no statistically significant differences, indicating that the difference in treatment cycles was not caused by an imbalance in initial disease severity or demographic features. However, there was a significant difference in the choice of chemotherapy regimen between the two groups (P=0.003). Patients who completed 8 cycles of treatment more frequently received the TCb-EC regimen (Full 8-cycle group: 31.3% vs. <8 cycles group: 6.4%). This suggests that the selection of specific chemotherapy regimens in clinical practice may be related to tumor biology and patient tolerance. Despite the aforementioned relationships between baseline characteristics and treatment strategy, the final treatment response and pathological outcomes did not show statistically significant differences between the groups: RCB 0 (indicating pathological absence of residual invasive cancer) (Full 8-cycle: 56.7% vs. <8 cycles: 44.7%, P=0.524); tpCR (Full 8-cycle: 56.7% vs. <8 cycles: 46.8%, P=0.297); bpCR (Full 8-cycle: 58.2% vs. <8 cycles: 51.1%, P=0.45). These results indicate that failure to complete all 8 cycles of immunotherapy did not significantly affect the pathologic response rate, suggesting a potential “early response” effect in neoadjuvant immunotherapy where the activated immune response may continue to exert antitumor effects even after treatment interruption.

**Table 1:** Comparison of baseline characteristics and treatment response by number of immunotherapy cycles.

Anti_PD1_times	<8	8	P.value
	(N=47)	(N=67)	
Anti_PD1_drug			0.737
Pembrolizumab	28(59.6%)	42(62.7%)	
Toripalimab	19(40.4%)	25(37.3%)	
Paltinum			0.427
no	26(55.3%)	32(47.8%)	
yes	21(44.7%)	35(52.2%)	
Chemo_regiem			0.003
EC-T	18(38.3%)	21(31.3%)	
EC-TCb	10(21.3%)	12(17.9%)	
others	9(19.1%)	2(3.0%)	
T-EC	7(14.9%)	11(16.4%)	
TCb-EC	3(6.4%)	21(31.3%)	
Age			0.677
>55	13(27.7%)	18(26.9%)	
≤35	5(10.6%)	11(16.4%)	
36~55	29(61.7%)	38(56.7%)	
Menopause			0.186
no	19(40.4%)	36(53.7%)	
yes	28(59.6%)	31(46.3%)	
T_stage			0.117
1	14(29.8)	13(19.4)	
2	27(57.4%)	50(74.6%)	
3	6(12.8%)	3(4.5%)	
4	0(0.0%)	1(1.5%)	
Nodal			0.595
negative	25(53.2%)	39(58.2%)	
positive	22(46.8%)	28(41.8%)	
RCB			0.524
0	21(44.7%)	38(56.7%)	
1	10(21.3%)	9(13.4%)	
2	7(19.1%)	13(19.4%)	
3	7(14.9%)	7(10.4%)	
tpCR			0.297
n	25(53.2%)	29(43.3%)	
y	22(46.8%)	38(56.7%)	
bpCR			0.45
n	23(48.9%)	28(41.8%)	
y	24(51.1%)	39(58.2%)	

RCB, Residual Cancer Burden; tpCR, Pathological Complete Response in Tumor; bpCR, Pathological Complete Response in Breast and Nodes; data are presented as “n (%)”, and intergroup comparisons were performed using the χ² test, with P<0.05 indicating a statistically significant difference.

### 3.2 Efficacy comparison of immunotherapy combined with platinum-based vs. non-platinum-based chemotherapy

To evaluate the potential additive effect of platinum drugs, patients were divided into two groups based on whether they received platinum-based chemotherapy: non-platinum group (N=58) and platinum group (N=56) (**Table 2**). A significant difference was observed in the choice of anti-PD-1 drug between the groups (P < 0.001). The non-platinum group had a higher proportion of toripalimab use (60.3%), whereas pembrolizumab dominated in the platinum group (83.9%). Regarding chemotherapy regimens, the non-platinum group primarily received EC-T (67.2%), while the platinum group mainly received EC-TCb (39.3%) and TCb-EC (42.9%), with a significant difference between groups (P < 0.001). Other baseline characteristics were balanced between the groups, showing no statistical difference (P > 0.05), including number of anti-PD-1 cycles, age, T stage, and nodal status, suggesting comparability in demographic and clinical features. Given the significant baseline differences in treatment strategy between groups, the results should be interpreted with caution. Under this limitation, no statistically significant differences were observed between the groups in RCB classification, tpCR, or bpCR (P > 0.05). The rate of RCB-0 (indicating complete or near-complete pathological response) was 50.0% in the non-platinum group and 53.6% in the platinum group (P=0.204). The tpCR rates were 51.7% and 53.6% (P=0.843), and bpCR rates were 56.9% and 53.6% (P=0.721), respectively. The above results indicate that despite significant differences in the composition of the treatment regimens between the two groups, within this limitation, we did not observe a statistically significant difference in the pCR rate between the two groups.

**Table 2:** Comparison of baseline characteristics and treatment response by use of platinum-based chemotherapy.

paltinum	no	yes	P.value
	(N=58)	(N=56)	
Anti_PD1_drug			
Pembrolizumab	23(39.7%)	47(83.9%)	<0.001
Toripalimab	35(60.3%)	9(16.1%)	
Chemo_regiem			
EC-T	39(67.2%)	0(0%)	<0.001
others	1(1.7%)	10(17.9%)	
T-EC	18(31.0%)	0(0%)	
EC-TCb	0(0%)	22(39.3%)	
TCb-EC	0(0%)	24(42.9%)	
Anti_PD1_times			
≤4	4(6.9%)	5(8.9%)	0.559
5~7	22(37.9%)	16(28.6%)	
8	32(55.2%)	35(62.5%)	
Age			
>55	19(32.8%)	12(21.4%)	0.283
≤35	9(15.5%)	7(12.5%)	
36~55	30(51.7%)	37(66.1%)	
T_stage			
1	17(29.3%)	10(17.9%)	0.105
2	39(67.2%)	38(67.9%)	
3	2(3.4%)	7(12.5%)	
4	0(0%)	1(1.8%)	
Nodal			
negative	35(60.3%)	29(51.8%)	0.357
positive	23(39.7%)	27(48.2%)	
RCB			
0	29(50.0%)	30(53.6%)	0.204
1	12(20.7%)	7(12.5%)	
2	13(22.4%)	9(16.1%)	
3	4(6.9%)	10(17.9%)	
tpCR			
n	28(48.3%)	26(46.4%)	0.843
y	30(51.7%)	30(53.6%)	
bpCR			
n	25(43.1%)	26(46.4%)	0.721
y	33(56.9%)	30(53.6%)	

Same as **Table 1**.

### 3.3 Significant impact of chemotherapy sequence on pathologic response rate

Patients were divided into 5 groups based on the different anthracycline-based sequential treatment regimens: EC-T (N=39), EC-TCb (N=22), T-EC (N=18), TCb-EC (N=24), and Other regimens (N=11) (**Table 3**). There were no significant differences in baseline characteristics such as age, menopausal status, T stage, or nodal status among the groups (P > 0.05), indicating comparability. Regarding pathological response, the distribution of RCB differed significantly among the groups (P=0.016). The TCb-EC group had the highest RCB-0 rate, reaching 79.2%. Further analysis showed that the tpCR rate also differed significantly among the groups (P=0.015), with the TCb-EC group having the highest tpCR rate (79.2%). Similarly, the bpCR rate showed significant intergroup differences (P=0.024), with the TCb-EC group achieving the highest bpCR rate (79.2%). This strongly suggests that the regimen sequencing taxane combined with platinum (TCb) before anthracyclines may maximize immune activation and tumor cell killing through synergistic effects, thereby providing the optimal pathological environment for achieving pCR.

**Table 3:** Clinicopathological characteristics and treatment response of breast cancer patients receiving different sequential anthracycline-based neoadjuvant treatment regimens.

Chemo_regiem	EC-T	EC-TCb	T-EC	TCb-EC	Others	P.value
	(N=39)	(N=22)	(N=18)	(N=24)	(N=11)	
Anti_PD1_drug						
Pembrolizumab	20(51.3%)	16(72.7%)	3(16.7%)	22(91.7%)	9(81.8%)	<0.001
Toripalimab	19(48.7%)	6(27.3%)	15(83.3%)	2(8.3%)	2(18.2%)	
Paltinum						
no	39(100%)	0(0%)	18(100%)	0(0%)	1(9.1%)	<0.001
yes	0(0%)	22(100%)	0(0%)	24(100%)	10(90.9%)	
Anti_PD1_times						
≤4	4(10.3%)	4(18.2%)	0(0%)	0(0%)	1(9.1%)	0.002
5~7	14(35.9%)	6(27.3%)	7(38.9%)	3(12.5%)	8(72.7%)	
8	21(53.8%)	12(54.5%)	11(61.1%)	21(87.5%)	2(18.2%)	
Age						
>55	12(30.8%)	7(31.8%)	6(33.3%)	4(16.7%)	2(18.2%)	0.619
≤35	8(20.5%)	3(13.6%)	1(5.6%)	3(12.5%)	1(9.1%)	
36~55	19(48.7%)	12(54.5%)	11(61.1%)	17(70.8%)	8(72.7%)	
Menopause						
no	18(46.2%)	8(36.4%)	10(55.6%)	12(50.0%)	7(63.6%)	0.597
yes	21(53.8%)	14(63.6%)	8(44.4%)	12(50.0%)	4(36.4%)	
T_stage						
1	13(33.3%)	3(13.6%)	4(22.2%)	5(20.8%)	2(18.2%)	0.619
2	25(64.1%)	16(72.7%)	13(72.2%)	16(66.7%)	7(63.6%)	
3	1(2.6%)	2(9.1%)	1(5.6%)	3(12.5%)	2(18.2%)	
4	0(0%)	1(4.5%)	0(0%)	0(0%)	0(0%)	
Nodal						
negative	22(56.4%)	10(45.5%)	12(66.7%)	16(66.7%)	4(36.4%)	0.324
positive	17(43.6%)	12(54.5%)	6(33.3%)	8(33.3%)	7(63.6%)	
RCB						
0	21(53.8%)	9(40.9%)	7(38.9%)	19(79.2%)	3(27.3%)	0.016
1	8(20.5%)	5(22.7%)	4(22.2%)	2(8.3%)	0(0%)	
2	8(20.5%)	3(13.6%)	5(27.8%)	2(8.3%)	4(36.4%)	
3	2(5.1%)	5(22.7%)	2(11.1%)	1(4.2%)	4(36.4%)	
tpCR						
n	17(43.6%)	13(59.1%)	11(61.1%)	5(20.8%)	8(72.7%)	0.015
y	22(56.4%)	9(40.9%)	7(38.9%)	19(79.2%)	3(27.3%)	
bpCR						
n	16(41.0%)	13(59.1%)	9(50.0%)	5(20.8%)	8(72.7%)	0.024
y	23(59.0%)	9(40.9%)	9(50.0%)	19(79.2%)	3(27.3%)	

Same as **Table 1**.

### 3.4 Efficacy comparison between toripalimab and pembrolizumab

The final analysis included 70 patients (61.4%) treated with pembrolizumab and 44 patients (38.6%) treated with toripalimab (**Table 4**). The two groups were well-balanced in most baseline characteristics but showed significant differences in chemotherapy regimen and platinum use (P < 0.001). Specifically, the pembrolizumab group used the TCb-EC regimen more frequently (31.4%), while the toripalimab group used the T-EC regimen more often (34.1%). Other variables, including age, menopausal status, T stage, nodal status, histological grade, hormone receptor status, HER2 status, and Ki67 expression level, showed no significant differences between the groups (P > 0.05). Regarding treatment response, the distribution of RCB grades showed no significant difference between the two groups (P=0.903). The RCB-0 rate (pCR) was 48.6% in the pembrolizumab group and 56.8% in the toripalimab group. The proportions of RCB-I, RCB-II, and RCB-III were also similar between the groups. The tpCR rate was 50.0% in the pembrolizumab group and 56.8% in the toripalimab group, with no statistically significant difference (P=0.777). The bpCR rate was 50.0% in the pembrolizumab group and 63.6% in the toripalimab group, with an overall rate of 55.3%; the intergroup difference was also not significant (P=0.362). In summary, although there were differences in some baseline characteristics, no significant intergroup differences were observed in the primary endpoints (RCB, tpCR, bpCR), suggesting that pembrolizumab and toripalimab exhibit comparable efficacy in the neoadjuvant setting and are similar in terms of pathological response.

**Table 4:** Comparison of baseline characteristics and treatment response between pembrolizumab and toripalimab groups.

Anti_PD1_drug	Pembrolizumab	Toripalimab	ALL	P.value
	(N=70)	(N=44)	(N=114)	
Chemo_regiem				
EC-T	20(28.6%)	19(43.2%)	39(34.2%)	<0.001
EC-TCb	16(22.9%)	6(13.6%)	22(19.3%)	
others	9(12.9%)	2(4.5%)	11(9.6%)	
T-EC	3(4.3%)	15(34.1%)	18(15.8%)	
TCb-EC	22(31.4%)	2(4.5%)	24(21.1%)	
Paltinum				
no	23(32.9%)	35(79.5%)	58(50.9%)	<0.001
yes	47(67.1%)	9(20.5%)	56(49.1%)	
Anti_PD1_times				
≤4	5(7.1%)	4(9.1%)	9(7.9%)	0.996
5~7	23(32.9%)	15(34.1%)	38(33.3%)	
8	42(60.0%)	25(56.8%)	67(58.8%)	
Age				
>55	19(27.1%)	12(27.3%)	31(27.2%)	0.979
≤35	11(15.7%)	5(11.4%)	16(14.0%)	
36~55	40(57.1%)	27(61.4%)	67(58.8%)	
Menopause				
no	33(47.1%)	22(50.0%)	55(48.2%)	0.957
yes	37(52.9%)	22(50.0%)	59(51.8%)	
T_stage				
1	18(25.7%)	9(20.5%)	27(23.7%)	0.567
2	43(61.4%)	34(77.3%)	77(67.5%)	
3	8(11.4%)	1(2.3%)	9(7.9%)	
4	1(1.4%)	0(0%)	1(0.9%)	
Nodal				
negative	33(47.1%)	31(70.5%)	64(56.1%)	0.0507
positive	37(52.9%)	13(29.5%)	50(43.9%)	
Grade				
2	34(48.6%)	19(43.2%)	53(46.5%)	0.854
3	36(51.4%)	25(56.8%)	61(53.5%)	
ER_status				
<1%	59(84.3%)	36(81.8%)	95(83.3%)	0.512
1%~10%	11(15.7%)	6(13.6%)	17(14.9%)	
>10%	0(0%)	2(4.5%)	2(1.8%)	
PR_status				
<1%	68(97.1%)	42(95.5%)	110(96.5%)	0.803
>10%	1(1.4%)	0(0%)	1(0.9%)	
1%~10%	1(1.4%)	2(4.5%)	3(2.6%)	
HER2_status				
0	19(27.1%)	12(27.3%)	31(27.2%)	0.889
1+	21(30.0%)	17(38.6%)	38(33.3%)	
2+	30(42.9%)	15(34.1%)	45(39.5%)	
Ki67_value				
>30%	57(81.4%)	36(81.8%)	93(81.6%)	0.999
≤30%	13(18.6%)	8(18.2%)	21(18.4%)	
RCB				
0	34(48.6%)	25(56.8%)	59(51.8%)	0.903
1	12(17.1%)	7(15.9%)	19(16.7%)	
2	13(18.6%)	9(20.5%)	22(19.3%)	
3	11(15.7%)	3(6.8%)	14(12.3%)	
tpCR				
n	35(50.0%)	19(43.2%)	54(47.4%)	0.777
y	35(50.0%)	25(56.8%)	60(52.6%)	
bpCR				
n	35(50.0%)	16(36.4%)	51(44.7%)	0.362
y	35(50.0%)	28(63.6%)	63(55.3%)	

Same as **Table 1**.

## 4 Discussion

This real-world analysis evaluated the efficacy of PD-1 inhibitors in the neoadjuvant treatment of breast cancer across four critical dimensions: treatment duration, chemotherapy regimen, administration sequence, and type of immune checkpoint inhibitor. The results demonstrated that neither completion of 8 cycles of immunotherapy nor the incorporation of platinum-based chemotherapy significantly influenced pathologic response rates. However, the sequencing of chemotherapy—particularly the TCb-EC regimen—was associated with a marked enhancement in efficacy. Notably, the domestically developed toripalimab exhibited comparable effectiveness to pembrolizumab on all primary efficacy endpoints. These findings offer valuable guidance for clinical decision-making regarding immune checkpoint inhibitor selection and combination strategy in neoadjuvant therapy.

First, regarding anti-PD-1 treatment cycles, this study identified no statistically significant difference in pCR rates between patients who completed 8 cycles of immunotherapy and those who did not. This finding challenges the prevailing view that completing the full course of neoadjuvant immunotherapy is essential for achieving clinical benefit (14, 15). The underlying mechanism may be attributed to the mode of action of immune checkpoint inhibitors: once T-cell-mediated immunity is activated, antitumor effects may persist and even induce an “immune memory” response over time, thereby attenuating the consequences of treatment interruption due to adverse events or patient choice (6, 16, 17). While this finding may appear inconsistent with the design of certain prospective trials—such as KEYNOTE-522, which mandated one year of treatment—it is important to emphasize that the EFS benefit observed in KEYNOTE-522 primarily resulted from adjuvant-phase continuation (18, 19). Our study, focused on pathological endpoints within the neoadjuvant setting, indicates that incompletion of the planned 8 cycles does not necessarily represent therapeutic failure. This insight carries important implications for the management of immune-related adverse events (irAEs), suggesting that clinicians may consider a risk-benefit assessment when encountering manageable toxicities rather than rigidly pursuing cycle completion, thus potentially preserving opportunities for achieving profound pathological responses.

Second, in comparing platinum-based and non-platinum-based regimens, our study—after adjusting for treatment cycles—found that the addition of platinum did not significantly increase the pCR rate. This result appears somewhat incongruent with the high pCR rates reported in the KEYNOTE-522 trial, which utilized a platinum-containing regimen (19). However, it is noteworthy that in the GeparNuevo study, the addition of platinum to a neoadjuvant regimen of paclitaxel plus durvalumab also did not significantly improve pCR rates (47.4% vs. 53.8%; p=0.7) (20, 21), which is consistent with our findings. Several factors may explain this discrepancy. First, population heterogeneity may play a role: KEYNOTE-522 enrolled only high-risk early-stage TNBC patients, whereas our study included all molecular subtypes. Hormone receptor-positive (HR+) patients may exhibit lower sensitivity to platinum, potentially diluting the overall treatment effect. Second, the synergistic mechanisms of immunotherapy may attenuate the observable benefit of platinum. Preclinical evidence indicates that platinum agents enhance antitumor immunity by inducing immunogenic cell death (ICD) (22–24); however, in the context of potent PD-1 inhibition, this incremental immunogenic effect may become less discernible (25). Third, the non-randomized design of our study may introduce selection bias, as more advanced cases might have been preferentially assigned to platinum-based regimens. Therefore, the universal additive value of platinum in immune–chemotherapy combinations remains uncertain and may depend heavily on specific tumor biology and treatment contexts.

Third, among the most compelling findings of this study is the pronounced effect of chemotherapy sequencing on treatment efficacy. The TCb-EC group (paclitaxel + carboplatin followed by epirubicin + cyclophosphamide) achieved a remarkable tpCR rate of 79.2%, significantly surpassing all other sequential approaches. This result is supported by a strong biological rationale. One plausible explanation involves an “immune priming” effect: both paclitaxel and carboplatin are known potent inducers of immunogenic cell death (ICD) (26–28). Initiating treatment with TCb may maximize tumor cell death, antigen release, and T-cell activation, thereby fostering an immunologically “hot” tumor microenvironment conducive to the subsequent administration of PD-1 inhibitors. Building upon this primed immune context, subsequent anthracycline-based chemotherapy (e.g., EC) may further amplify antitumor responses and mitigate immunosuppressive cell populations (13, 29), culminating in synergistic efficacy and increased pCR rates. This observation is consistent with emerging concepts regarding the timing of chemo-immunotherapy integration. For instance, although the IMpassion031 (30) and KEYNOTE-522 (14, 15) trials adopted a fixed EC-T sequence followed by taxane/platinum, their elevated pCR rates also underscore the importance of early immune engagement. Our results refine this notion by suggesting that initiating therapy with a highly immunogenic regimen (such as TCb) may be instrumental in eliciting profound antitumor responses in the context of immunotherapy. These findings provide pertinent real-world evidence for optimizing combination strategies in neoadjuvant immuno-chemotherapy and propose that “front-loading immunogenic chemotherapy” may represent a key strategic principle worthy of validation in prospective clinical trials.

Finally, and central to the conclusion of this study, toripalimab demonstrated efficacy comparable to pembrolizumab in the neoadjuvant setting for breast cancer. Although some baseline characteristics differed between groups (e.g., preference for certain chemotherapy regimens), statistical analyses showed no significant differences in key efficacy endpoints, including RCB classification, tpCR, and bpCR. These results strongly support the value of toripalimab—a domestically developed innovative agent—in the treatment of breast cancer. Although head-to-head randomized controlled trials are still lacking, our real-world findings align with efficacy trends observed between toripalimab and pembrolizumab in other malignancies, such as nasopharyngeal carcinoma, lung cancer, and colorectal cancer (31–33). While pembrolizumab has become a global standard of care based on trials like KEYNOTE-522, its high cost and reimbursement barriers limit its accessibility in many settings (34, 35). In recent years, there has been growing interest in identifying more cost-effective immunotherapy approaches. For example, the NeoTENNIS study previously reported promising efficacy with toripalimab in neoadjuvant TNBC, with a pCR rate of 55.7% (13). Through rigorous statistical methodologies, including Chi-square tests, Fisher’s exact tests, our study provides compelling real-world evidence supporting the comparable efficacy of toripalimab relative to pembrolizumab. This finding is particularly relevant for regions with constrained healthcare resources, offering an effective and accessible therapeutic alternative that may help advance equity in breast cancer care.

This study has several limitations. Its single-center, retrospective design inherently introduces potential selection bias and limits the generalizability of the findings. The relatively small sample size, though substantive, may reduce statistical power, particularly in subgroup analyses. Furthermore, the sample size and the distribution of outcomes across multiple subgroups precluded a reliable multivariate logistic regression analysis to adjust for potential confounders and identify independent predictors of pCR. Consequently, our findings regarding the impact of chemotherapy sequence should be interpreted as associative rather than causative, and future validation in larger cohorts is needed. The absence of long-term survival outcomes such as event-free survival (EFS) or overall survival (OS) restricts the ability to correlate pCR with ultimate clinical benefit. Additionally, important biomarker information such as PD-L1 expression and tumor-infiltrating lymphocyte (TIL) levels was not systematically analyzed, which weakens the biological interpretation of response mechanisms and limits the ability to identify patient subsets most likely to benefit from treatment.

In conclusion, this real-world study indicates that the domestically developed PD-1 inhibitor toripalimab exhibits efficacy comparable to pembrolizumab in the neoadjuvant treatment of breast cancer. The sequencing of chemo-immunotherapy—particularly the preferential use of the TCb-EC regimen—was identified as a critical factor associated with improved pCR rates, whereas the number of treatment cycles and incorporation of platinum did not significantly influence pathological outcomes. Future large-scale, prospective, and biomarker-driven studies are warranted to validate these findings and refine optimal therapeutic strategies, ultimately advancing precision and individualized immunotherapy in breast cancer.

## Data Availability

The original contributions presented in the study are included in the article/supplementary material. Further inquiries can be directed to the corresponding authors.
